# An analysis of inclusive education practices in East Java Indonesian preschools

**DOI:** 10.3389/fpsyg.2023.1064870

**Published:** 2023-02-16

**Authors:** Mohd Hanafi Mohd Yasin, Sinta Yuni Susilawati, Mohd Mokhtar Tahar, Khairul Azhar Jamaludin

**Affiliations:** ^1^Faculty of Education, University Kebangsaan Malaysia, Bangi, Selangor, Malaysia; ^2^Fakultas Ilmu Pendidikan, Universitas Negeri Malang, Malang, Jawa Timur, Indonesia

**Keywords:** inclusive education, index for inclusion, inclusive cultures, preschools, East Java

## Abstract

Poor access to quality education among preschool students in Indonesia is a cause for concern. To address this issue, the first step is to identify the current level of inclusive education practises in these institutions. Thus, this study is aimed at identifying the level of inclusivity of Indonesian preschools, particularly in East Java, from the perspective of education practitioners. This study employed a sequential explanatory mix design. A survey questionnaire and semi-structured interviews were utilised in collecting the data. A total of 277 education practitioners, including principals and teachers at the preschool level, were randomly sampled to answer the questionnaire. As interview respondents, 12 teachers and principals were recruited *via* purposive sampling. Generally, the findings indicated that community building for inclusive education was found to be at an average level (*M* = 3.418, SD = 0.323), whereas building inclusive values in preschools was found to be at a high level (*M* = 4.020, SD = 0.414). In support of this, the findings of the semi-structured interviews suggested that the school community was aware of the differences among students and that respecting each other was generally practised among the school community. However, poor community involvement to support inclusive education was a challenge in most Indonesian preschools. These findings are crucial for stakeholders and policymakers to continue promoting community awareness and supporting inclusive education in these institutions.

## 1. Introduction

Recognizing the right of all children to education is ensuring inclusion and equity in education. The United Nations Educational, Scientific, and Cultural Organization (UNESCO, 2017) defines inclusion as the process of assisting children to overcome barriers and challenges to quality education, and equity as the efforts to foster a sense of fairness so that all students are treated equally in educational settings. Clearly, inclusive education is a basic human right and the foundation for accomplishing social cohesion (UNESCO, 2005, 2008). This implies that educational institutions should value the diversity, achievement, and participation of all students, strategize planning to enhance their participation and cater to their specific needs, continuously promote understanding and awareness of inclusive and equitable education, and involve stakeholders and the community in its implementation (UNESCO, 2017). Although many educational institutions have supported inclusive education in recent years (Kyriakides et al., 2018), it remains difficult to implement and maintain its quality.

Inclusion is seen as beneficial to both individual children and society as a whole [United Nations Educational, Scientific, and Cultural Organization (UNESCO), 1994, 2020; Booth and Ainscow, 2002; Odom et al., 2004; Florian and Black-Hawkins, 2011; Mitchell, 2014; Barton and Smith, 2015; Collett, 2018; European Commission, 2020); and is a Sustainable Development Goal to be achieved by 2030 (United Nations, 2015b, Goal 4, Target 4.5]; in Johanna Lundqvist, 2030 The Education Initiative 2030 is a significant step forward, where the priority is to guarantee that everyone receives a high-quality education and provide opportunities for lifelong learning for all (United Nations, 2015a). How inclusion could be put into practise is an important question to answer. “The current debate is not about what inclusion is or why it is necessary; the key question is how it can be achieved.”

The quality of early childhood education is a major concern for the government, and it has recently become a priority for many international and European organizations, including the Organization for Economic Cooperation and Development (OECD), the United Nations Educational, Scientific and Cultural Organization (UNESCO), and the Organization for Economic Cooperation and Development (OECD). UNESCO, UNICEF, the European Commission, Eurydice, and the European Agency for Special Needs and Inclusive Education International organisations have recently emphasised the importance of prioritising high-quality early childhood education and parenting in order to eliminate inequalities in lifelong learning [European Agency for Special Needs and Inclusive Education (European Agency), 2017].

In Indonesia, inclusive education aims to solve a variety of educational issues for children with special needs through learning activities in regular schools that provide positive values for student skills. Preschool inclusive education can help children prepare for adulthood (Sakti, 2020). According to Jumiatin et al. (2020), if the implementation is done correctly, the inclusive education service system that has been in place since preschool will be able to contribute to the education stage of children with special needs in the next education stage. This is certainly crucial to the purpose of education in Indonesia, where the education system should always provide maximum access and convenience for the citizens to learn regardless of their differences and backgrounds. Early childhood education institutions throughout Indonesia are continuing to develop and implement inclusive education.

The Index for Inclusion (Ainscow and Booth, 2011) is an example of a self-development evaluation tool specifically designed to support and assist the process of developing inclusive education. It is intended to support critical reflection and action through a process of review and self-evaluation, and it could be modified for local use and adapted to meet the needs of individual agencies. Braunsteiner (2016) cites EASPD (2012). Early in the school development process, three dimensions must be considered: culture, policy, and practice. Each dimension is split into two sections. As planning frameworks, both dimensions and sections can be used (Booth and Ainscow, 2016, p. 13). The inclusion index measures the success of preschool inclusive education implementation. The purpose of this research is to better understand what factors promote inclusive education implementation in terms of the cultural dimension in the context of preschool education.

## 2. Methodology

### 2.1. Research design

The current study employed a sequential explanatory mix design, beginning with quantitative data collection and continuing with qualitative data collection (Creswell, 2009; Sugiyono, 2019). McBride et al. (2019) assert that this design is beneficial in ensuring a reliable and credible source of research data as its emergent approach expands the findings of the quantitative data to further understanding the studied issues. Therefore, in this study, a survey questionnaire was first administered on the research participants and semi-structured interviews were conducted to validate, deepen, and broaden the quantitative data (Creswell and Plano Clark, 2018; Sugiyono, 2019).

### 2.2. Participants

Participants for this study were selected using random stratified sampling technique. Four categories of respondents (principals, special education teachers, school administrators, and teachers) were identified from a population of 427 education administers and practitioners in East Java Preschools. A minimum of 205 respondents are required as the minimum sampling size for the population of 440 individuals (Krejcie and Morgan, 1970). Thus, 206 participants were identified, and these participants were representatives of all strata. The number of participants for each stratum was calculated using the following formula [as suggested by Er (2012)]:

Participants for a selected stratum = (Number of required participants/population) × total of participants in strata.

Based on the above formula, the total number of participants for each stratum is as the following:

1. Principals = 13 out of 26 principals.
2. Special education teachers = 13 out of 26 special education teachers.
3. School administrators = 21 out of 44 school administrators
4. Teachers = 159 out of 220 teachers.

On the other hand, a total of 12 respondents from these four categories were selected as the interview participants. These respondents were selected based on the following criteria:

1. have obtained a diploma/degree in the field of preschool education.
2. have more than 5 years of working experience in the special preschool education.

### 2.3. Instrument

The survey questionnaire was adapted from Index for Inclusion, Creating an Inclusive Culture and used a 5-Likert scale (from 1 for strongly disagree to 5 for strongly agree; Creswell, 2002; Ainscow and Booth, 2011). There were three sections in this questionnaire: Section A: Demographic Information, Section B: Community Development, and Section C: Inclusive Value Development. Table 1 provides a summary of this questionnaire.

**Table 1:** Questionnaire items.

Section	No. of question
Section A: Demographic information	4
Section B: Community development	9
Section C: Inclusive value development	9
Total	22

To support the quantitative data, the interview protocol was developed to further understand the community development efforts and inclusive values development among education practitioners in inclusive preschools. The questions focussed on the factors that can help promote inclusive education, and how was current practice of inclusive education in these selected schools (based on two aspects: community development and inclusive value development).

### 2.4. Data collection and analysis

The data collection was held at the participant’s institution. Permission to conduct this research was obtained from the Ministry of Education, Culture, Research, and Technology Indonesia before any data was collected. The participants were asked to sign a consent form and were informed about the purpose of the study, their roles as participants, and the confidentiality of their responses.

For analysis, the quantitative data was analyzed using descriptive statistics - the frequency, mean, and standard deviation as the questionnaire has an interval scale and measurement ratio (Hair et al., 2007). Furthermore, the mean values were interpreted according to Jamil (2002) recommendation: 1.00–2.33 represents a low level of integration, 2.34–3.66 represents an average level of integration, and 3.67–5.00 represents a high level of integration. On the other hand, the qualitative data was transcribed and analyzed thematically, based on Braun and Calrke (2006) six-step analysis – starting with familiarizing with the data, and is followed by generating initial codes, identifying themes, revising the identified themes, defining and naming the themes, and writing the report.

## 3. Findings

### 3.1. Resource identification initiative

Table 2 is a summary of the respondents’ demographic information. Generally, most of the respondents hold a bachelor’s degree (*n* = 197, 71.12%). From a total of 277 respondents, 175 respondents (63.18%) completed their respective degrees in preschool education field, and 231 (83.39%) of them have working experience between five to less than 10 years.

**Table 2:** Demographic information.

Item	Frequency	Percentage
Education level		
Doctoral degree	0	0
Master’s degree	80	28.88
Bachelor’s degree	197	71.12
Total	277	100.00
Field of education		
Inclusive education	21	7.58
Preschool education	175	63.18
Others	81	29.24
Total	277	100.00
Teaching experience		
0 to less than 5 years	0	0
5 to less than 10 years	231	83.39
10 to 15 years	46	16.61
Total	277	100.00

Table 3 illustrates the results of mean score analysis of the level of inclusive education practices in East Java preschools. This indicates that the overall level of inclusive education practices was at a high level. In addition, inclusive value development achieved a higher mean score (*M* = 4.020, SD = 0.424). However, community building achieved a slightly lower mean score (*M* = 3.418, SD = 0.323), indicating an average level of integration.

**Table 3:** The mean scores for community building, and inclusive values development.

Item	*M*	*SD*	Interpretation
Community building			
A1: Everyone is welcome.	3.116	0.565	Average
A2: Students help each other.	3.112	0.556	Average
A3: Staff and students respect each other.	4.520	0.657	High
A4: Staff and parents/guardians work with each other.	2.975	0.477	Average
A5: Staff and administrators have good cooperation.	4.500	0.635	High
A6: School as a model of democratic citizenship.	3.126	0.733	Average
A7: Adults and children respond to various gender roles.	2.097	0.372	Low
A8: School and local communities thrive together.	3.036	0.509	Average
A9: The staff relates what happens at school to the students’ daily lives at home.	4.270	0.575	High
Total	3.416	0.564	Average
Inclusive values development			
B1: School develops inclusive values that are shared together.	4.160	0.673	High
B2: School encourages everyone to respect all forms of human rights	4.330	0.685	High
B3: School and local community work together to develop inclusive values.	3.148	0.688	Average
B4: Inclusiveness is seen as increasing participation for everyone.	4.190	0.572	High
B5: Students are valued equally.	3.043	0.606	Average
B6: Students are treated equally.	4.200	0.809	High
B7: School avoids any form of discrimination.	4.460	0.616	High
B8: School encourages interaction and non-violent resolution of arguments.	4.390	0.565	High
B9: Schools encourage students and adults to feel good about themselves.	4.250	0.698	High
Total	4.019	0.656	High

The overall mean score for Community Building was *M* = 3.416 (SD = 0.564), indicating an average level of integration. However, three items achieved a high level of integration, indicating that the respondents believed they respect each other (*M* = 4.520, SD = 0.657), have a good cooperation (*M* = 4.500, SD = 0.635), and staff relates what happens at school to the students’ daily lives at home (*M* = 4.270, SD = 0.575). This was supported with the interview responses as the participants indicated that they respect, cooperate and relate students’ prior experience at home to school:

We respect one another. We always work together with all elements involved in education implementation. (PSR1)

We definitely have mutual respect. We always work together as well. (GAR2)

Of course, we respect one another. We also collaborate to achieve inclusive education. (GPR4)

It needs to be noted that we must respect one another. We also rely on one another and collaborate. (KTR4)

Interestingly, it was found that responding to different gender roles was at the lowest level of integration (*M* = 2.097, SD = 0.372). A further analysis from the interview indicated that it was difficult to properly respond to this difference as the current practices were still poor and school was not encouraging to acknowledge and celebrate differences. For instance, the respondents stated that:

We have not been able to accept all students indeed, what we accept is still limited considering the limitations we have. I am afraid that they will not be served well if all are accepted. We are also still learning in every way to implement this inclusive education. (PSR4)

The school does not have the courage to accept all students. (GAR3)

Because of the limitations that we have for now, we initially limit the number of students with special needs that we accept.

On the other hand, the overall mean score for Inclusive Value Development was *M* = 4.019 (SD = 0.373), indicating a high level of integration. The respondents supported that:

All of us do our best to always provide needed services for all children, including special needs children. (PSR1)

Undoubtedly, students in general were valued equally as the school encouraged inclusivity in teaching and learning activities:

We work very hard to respect each student's rights and to be non-discriminatory, to involve all students in learning, to serve their learning needs, to solve problems together, and to interact well with one another. (PSR.1)

To be able to realize a friendly school, it must begin by respecting the rights of all students and respecting differences. What I do is non-discriminatory, and I try to provide the best service possible as needed, and all problems are properly resolved. (GAR. 2)

The significance of achieving education for all by respecting and not discriminating against the rights of all students. I make every effort to meet the learning needs of students with special needs. If there are any issues, particularly with learning, we will consult with all relevant parties to find the best solution. (GPR. 2)

The most important thing is to respect the rights of every student, to respect difference, to be accommodating, not discriminatory, and to solve problems in a positive way. (KTR.2)

However, two items achieved an average level: School and local community work together to develop inclusive values (*M* = 3.148, SD = 0.688), and students are valued equally (*M* = 3.043, SD = 0.606). This is because they respondents expressed that community involvement was limited and thus, affecting the effort to collaboratively develop inclusive values. They stated that:

Community involvement is limited to the participation in school committee activities. (PSR1)

Our community collaboration remains limited because not all ordinary people understand and care about the education of students with special needs. (GAR4)

Community involvement is limited to committee activities, with the exception of related experts in the form of crew member identification. (GPR.4)

Cooperation remains restricted to committee activities. (KTR2)

## 4. Discussion

Undeniably, the preschools in East Java have attempted to implement inclusive education by practicing community building and inclusive values development at different levels – from the administrators to teachers and local community. However, in this study, it was found that community building was still at an average level (*M* = 3.416, SD = 0.564).

To successfully develop a community that is sensitive and responsible for inclusive education, the school’s ideology needs to be refined. Thus, shared values, beliefs, and perceptions that are mutually shared by each community member should be emphasised (Hatzipanagiotou, 2008; Schein and Schein, 2017). Alexaki et al. (2022) strongly believe that access and practises under the inclusive policy are typically treated as a separate unit. As a result, simply having access is insufficient if the practises in these preschools are subpar. In this study, this might be the reason. A closer identification of the findings indicated that responding to different gender roles was at the lowest level of integration (*M* = 2.097, SD = 0.372). The interview findings also supported the fact that the current practise was not responsive to this difference.

On the other hand, the inability of teachers, staff, parents, and the community in general to address differences and avoid discrimination against individual differences is still a problem in the context of Indonesian education. One of the barriers to effective implementation of inclusive education in Indonesia is teacher competency (Tarmansyah, 2009; Yusuf and Yeager, 2011; Ministry of Education and Culture Indonesia, 2019). As a result, these students continue to face challenges in society, particularly in educational settings.

This, however, will always go well if the existing system is continuously improved. In addition, the school works to improve learning quality by emphasising the importance of student cooperation. Cooperation among students is essential in community development as part of the effort to implement inclusive education (Sánchez et al., 2019).Peer-to-peer learning, on the other hand, has been shown to improve academic outcomes and school inclusion (Sánchez and Dez, 2013), as evidenced by the findings of the European project (Included Ed., 2006–2010), as elaborately discussed by Sánchez et al. (2019). As a result, in order to realise the implementation of inclusive education, student collaboration must be developed. Parental involvement in learning has so far been limited to broad issues. As a result, as a barrier to achieving inclusive education implementation, schools should attempt to involve parents more in the planning, implementation, and assessment processes. According to Sánchez et al. (2019), one of the barriers to quality and inclusive education is a lack of collaboration or involvement of families and teachers in the implementation of inclusive education. Of course, if collaboration is not viewed as a critical component of school performance, it becomes more difficult to create an inclusive culture. Schools should also strengthen their relationships with the surrounding community in order to develop and implement inclusive education collaboratively, and each gender’s role should be well understood and communicated across the community members.

On the other hand, it was found that inclusive value development was at a high level of integration (*M* = 3.719, SD = 0.373). These preschools have taken initiatives to achieve this, especially trying to accept children without discrimination, even though, due to limitations, the school has not been able to fully accept all children with various needs and abilities (Efendi, 2011). Furthermore, the school strives to meet each student’s learning needs by implementing child-friendly learning. In order to create inclusive schools, all elements involved in the implementation of inclusive education must collaborate and solve existing problems in constructive ways.

The findings provide a clearer indication that the East Java preschools are practising inclusive values. Specifically, inclusive value development, encouragement to respect each other, increased participation, equal treatment for all, and appreciating oneself are pivotal to inculcating inclusivity and equity in education (UNESCO, 2017). As a result, much can be learned and replicated at other levels of education in Indonesia from how and what is practised in these institutions. A closer examination of the findings revealed an average level of engagement between the community and the local community, as well as how students are valued equally. This calls for room for improvement in ensuring the current practises can be improved by involving the local community, especially parents, to help promote and enhance inclusivity in schools. This resonates with the findings by Lundqvist (2022), who similarly highlighted the importance of community involvement in developing a more inclusive and equitable learning environment for preschool children.

Undeniably, schools are still limited in their ability to involve the community in order to develop inclusive education (Efendi, 2011). Suhendri (2020) suggested that awareness should be developed among community members to support inclusive education practices. It is critical to provide adequate information to the general public. Undeniably, the use of social media platforms is pivotal to addressing this problem. Suhendri (2020) believed that a systematic campaign and awareness programmes should be developed to ensure the message is reaching the target group.

## 5. Conclusion

Generally, the findings of this study have provided insight on the current inclusive education practises in East Java preschools. Education should be responsive to the needs and differences of children, particularly those with special needs. As emphasised by UNESCO (2017), the obstacles and challenges faced by these children should be strategically addressed, with a particular emphasis on increasing the awareness and participation of teachers, administrators, and the general public in order to ensure its effective implementation. This study provided insightful findings for policymakers, educators, and society on the level of inclusive integration in East Java preschools. Even though inclusive value development is at a high level, there is still a need to raise awareness about student differences and ideal inclusive education practices. Furthermore, immediate action should be taken to ensure that community building for inclusive education is at a higher level. Interestingly, the findings indicated that community building was at an average level. It is important to note that without strong community development, efforts to ensure that every student is valued and that different needs are strategically addressed will fall short.

Future researchers are encouraged to expand on the current study by identifying parents’ perspectives on inclusive education practises in East Java preschools. As an important part of a society, their perspectives, voices, and experiences are critical to better understanding the strengths and challenges of implementing inclusive education in these institutions. It is also suggested that a comparison of participants’ perceptions of the level of implementation of inclusive education based on their location, job type, and other social demographic characteristics be conducted to further investigate the differences in their opinions and practices. This information is critical for improving current preschool practises in East Java.

## Data availability statement

The original contributions presented in the study are included in the article/supplementary material, further inquiries can be directed to the corresponding author.

## Ethics statement

The studies involving human participants were reviewed and approved by the Ministry of Research, Technology and Higher Education Indonesia. The participants provided their written informed consent to participate in this study.

## Author contributions

MY, SS, and MT: conceptualization and data collection. MY, SS, and KJ: writing, review, and editing. SS and KJ: data analysis. All authors contributed to the article and approved the submitted version.

## Conflict of interest

The authors declare that the research was conducted in the absence of any commercial or financial relationships that could be construed as a potential conflict of interest.

## Publisher’s note

All claims expressed in this article are solely those of the authors and do not necessarily represent those of their affiliated organizations, or those of the publisher, the editors and the reviewers. Any product that may be evaluated in this article, or claim that may be made by its manufacturer, is not guaranteed or endorsed by the publisher.
